# An R package for an integrated evaluation of statistical approaches to cancer incidence projection

**DOI:** 10.1186/s12874-020-01133-5

**Published:** 2020-10-15

**Authors:** Maximilian Knoll, Jennifer Furkel, Jürgen Debus, Amir Abdollahi, André Karch, Christian Stock

**Affiliations:** 1 Department of Radiation Oncology, Heidelberg University Hospital, Im Neuenheimer Feld 400, 69120 Heidelberg, Germany; 2 Faculty of Biosciences, Heidelberg University, Heidelberg, Germany; 3 Clinical Cooperation Unit Radiation Oncology, German Cancer Research Center (DKFZ), Im Neuenheimer Feld 280, 69120 Heidelberg, Germany; 4 German Cancer Consortium (DKTK) Core Center Heidelberg, Heidelberg, Germany; 5 Institute of Epidemiology and Social Medicine, University of Muenster, Albert-Schweitzer-Campus 1, 48149 Muenster, Germany; 6 Institute of Medical Biometry and Informatics (IMBI), University of Heidelberg, Im Neuenheimer Feld 130.3, 69120 Heidelberg, Germany; 7 Division of Clinical Epidemiology and Aging Research, German Cancer Research Center (DKFZ), Heidelberg, Germany

**Keywords:** Cancer epidemiology, age-period-cohort model, Bayesian model, Cancer incidence projection, INLA

## Abstract

**Background:**

Projection of future cancer incidence is an important task in cancer epidemiology. The results are of interest also for biomedical research and public health policy. Age-Period-Cohort (APC) models, usually based on long-term cancer registry data (> 20 yrs), are established for such projections. In many countries (including Germany), however, nationwide long-term data are not yet available. General guidance on statistical approaches for projections using rather short-term data is challenging and software to enable researchers to easily compare approaches is lacking.

**Methods:**

To enable a comparative analysis of the performance of statistical approaches to cancer incidence projection, we developed an R package (incAnalysis), supporting in particular Bayesian models fitted by Integrated Nested Laplace Approximations (INLA). Its use is demonstrated by an extensive empirical evaluation of operating characteristics (bias, coverage and precision) of potentially applicable models differing by complexity. Observed long-term data from three cancer registries (SEER-9, NORDCAN, Saarland) was used for benchmarking.

**Results:**

Overall, coverage was high (mostly > 90%) for Bayesian APC models (BAPC), whereas less complex models showed differences in coverage dependent on projection-period. Intercept-only models yielded values below 20% for coverage. Bias increased and precision decreased for longer projection periods (> 15 years) for all except intercept-only models. Precision was lowest for complex models such as BAPC models, generalized additive models with multivariate smoothers and generalized linear models with age x period interaction effects.

**Conclusion:**

The incAnalysis R package allows a straightforward comparison of cancer incidence rate projection approaches. Further detailed and targeted investigations into model performance in addition to the presented empirical results are recommended to derive guidance on appropriate statistical projection methods in a given setting.

## Background

Projection of future cancer incidence is an important task in cancer epidemiology. The results are of interest also for biomedical research and public health policy. In particular, cancer prevention and screening programs require reliable estimates of future cancer incidence to allow informed decisions on their design and to facilitate evaluations [1, 2]. Projections are often performed using long-term data (> 20 yrs) from population-based cancer registries [3]. For short-term data, there appears to be a lack of guidance which statistical approach to choose. The need to base projection models on relatively short-term data is relevant e.g. for Germany, where aggregated data of cancer incidence on a national level is only available from 1999 on, as well as for many countries with newly established cancer registries. Even though it might be challenging to give general guidance on which projection approach to choose, software enabling comparison of multiple competing methods for a given research question might prove useful, but flexible, extensive and easy to use tools are missing.

A selection of previously applied projection models is outlined in [4]. Relatively simple approaches assuming constant rates were utilized [5, 6], as well as more complex age-period (AP) models formulated as generalized linear models (GLMs) with or without interaction effects [7–9]. Clements et al. use generalized additive models (GAMs) [10]. GAMs can include uni- or multivariate smoothers in their linear predictors. An established model class for incidence projections based on long-term observation data are age-period-cohort (APC) models, which additionally incorporate a cohort effect [11, 12]. Even though projections of APC usually yield robust results, the APC identification problem impairs direct interpretability of single effects [13, 14].

Projection models are often fitted within a classical maximum likelihood (ML) or restricted maximum likelihood (REML) framework [15–17]. Alternatively, a Bayesian framework may be used [18, 19]. Bayesian model estimation can be implemented using Markov-Chain Monte Carlo (MCMC) methods, which are computationally intensive. A recently developed computationally far less demanding alternative is Integrated Nested Laplace Approximation (INLA) [20, 21].

GAMs usually incorporate splines to fit univariate trends or tensor product smoothers for multivariate trends (i.e. interactions between function of continuous variables). In the classical frequentist framework, such models can be fit e.g. using the mgcv-package in R [22]. Uni- and multivariate smoothers can directly be incorporated into the model formula, e.g. as splines or tensor product smoothers.

Recently, a highly flexible Bayesian APC (BAPC) model based on the INLA approach has been proposed for future cancer incidence projections which assumes a Poisson distribution of incidence counts [19]. Havulinna et al. demonstrate that interactions between effects can be modeled by specifying appropriate priors [18].

Given the lack of guidance on statistical modeling approaches to cancer incidence projection and the increasing understanding across sciences that neutral comparisons of statistical methods are needed [23–25], we developed an R package which allows an integrated comparison of model performance metrics in the above described context. We thereby aim to facilitate an informed choice of statistical models and the development of methodological guidance. Due to the desirable flexibility in modeling options and the probabilistic interpretation of results in a Bayesian framework as well as the computationally efficient implementation, we emphasize the INLA approach. To demonstrate the functionality of the new package we provide an extensive empirical benchmarking analysis of a selection of potentially applicable modeling approaches using observed long-term data from three population-based cancer registries.

## Methods

### Cancer registry data

Three low incident tumor sites/entities (brain tumors, kidney cancer, melanoma) and four high incident entities (lung, breast, colorectal, prostate) were selected from three population-based cancer registries: SEER-9 [26], NORDCAN [27] and Saarland [28]. Incidence data of patients below the age of 20 yrs. and older than 84 years (available only as aggregated data) were excluded from analysis. Specific selection criteria are shown in Table 1. Data was separately analyzed for males and females with few exceptions (prostate cancer: only male; breast cancer: only female in the Saarland data, males and females in SEER-9 and NORDCAN data). A representative data structure of incidence and population data, as also used in the incAnalysis package, is shown in Suppl.-Tbl. 1. Cancer cases (incidence) for a given year are stored in rows (most recent year in bottom row) and each row is separated by age(−group) in columns in increasing order from left to right.

**Table 1:** Selection details for analyzed tumor sites/entities for the three cancer registries and selected incidence data. ^−^low, ^+^high incidence. ^1^: male/female; age 60 for SEER-9 and age group 60–64 otherwise

registry	entity/site	selection	#cases^**1**^	
			**1990**	**2014**
SEER-9	Glioblastoma^−^	HISTO3V: 9440	13/7	21/14
	Kidney cancer^−^	PRIMSITE: C649	35/23	99/45
	Melanoma^−^	PRIMSITE: C440–449	54/36	227/144
	Lung and bronchial tumors^+^	PRIMSITE: C340–349	263/169	221/183
	Breast cancer^+^	PRIMSITE: C500–509	4/404	2/845
	Colorectal cancer^+^	PRIMSITE: C18–20	151/100	182/120
	Prostate cancer^+^	PRIMSITE: C61.9	250	595
NORDCAN	Brain, central nervous system^−^	cancer: 340	194/192	299/309
	Kidney^−^	cancer: 290	182/118	343/158
	Melanoma of skin^−^	cancer: 310	182/157	551/447
	Lung^+^	cancer: 180	929/411	906/900
	Breast^+^	cancer: 200	9/1314	21/2877
	Colorectal^+^	cancer: 590	638/551	1148/872
	Prostate^+^	cancer:261	762	3878
Saarland	Brain tumors [Gehirn] ^-^	loc: 191	2/2	8/2
	Kidney cancer [Niere, sonst.u.n.n.bez. Harnorgane] ^-^	loc: 189	18/9	13/6
	Melanoma [Bösartiges Melanom der Haut] ^-^	loc: 712	3/6	15/10
	Lung, bronchial and tracheal tumors [Luftröhre, Bronchien u. Lunge] ^+^	loc: 162	109/14	86/46
	Female breast tumors [Weibliche Brustdrüse] ^+^	loc: 174	75	107
	Colorectal cancer [Dick- und Mastdarm] ^+^	loc: 153 + 154	47/49	59/36
	Prostate cancer [Prostata] ^+^	loc: 185	36	95

From the Surveillance, Epidemiology, and End Results (SEER) Program in the United States, SEER-9 cancer incidence data (1973–2014) were accompanied by population data, available in 1 year age groups.

NORDCAN data (1960–2015) comprise cancer incidence data from Denmark, Finland, Iceland, Norway, Sweden, Faroe Islands and Greenland. The data were retrieved from the NORDCAN website on 2018-08-01. Incidence data were available in 5 year age groups. Population matrices were calculated from the person-years at risk information.

Cancer incidence data from Saarland (1970–2014), a German federal state with a long-established cancer registry, were obtained from the Saarland cancer registry website on 2018-08-01 (5 yr age groups). Population data were retrieved from the health report system of the federal government (up to 2012) und from the website of Saarland for the years 2013/14 [29, 30].

### Projection models

**Fig. 1:**
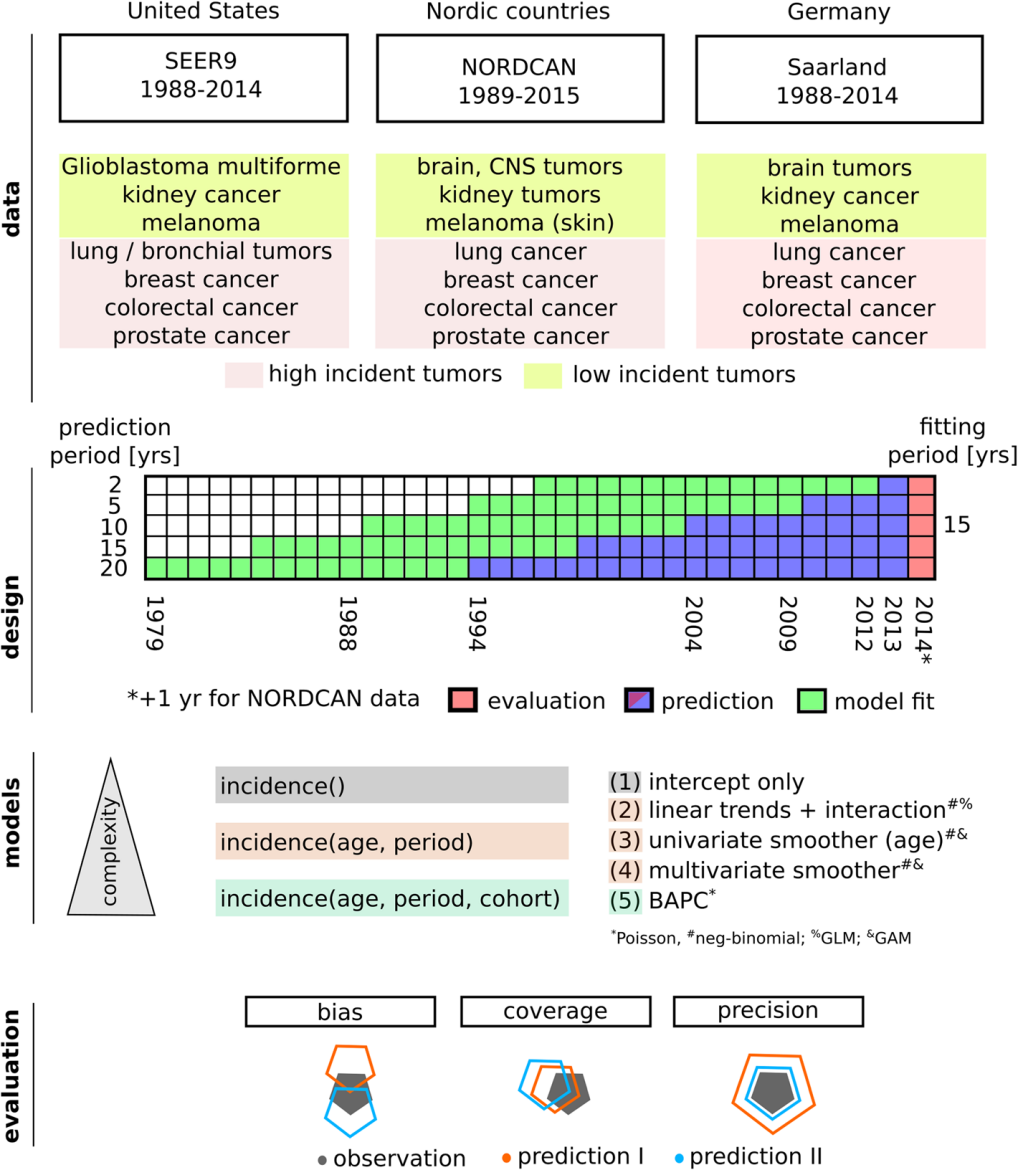
Overview of the analyzed cancer registry data, study design, model selection and evaluation metrics

**Fig. 2:**
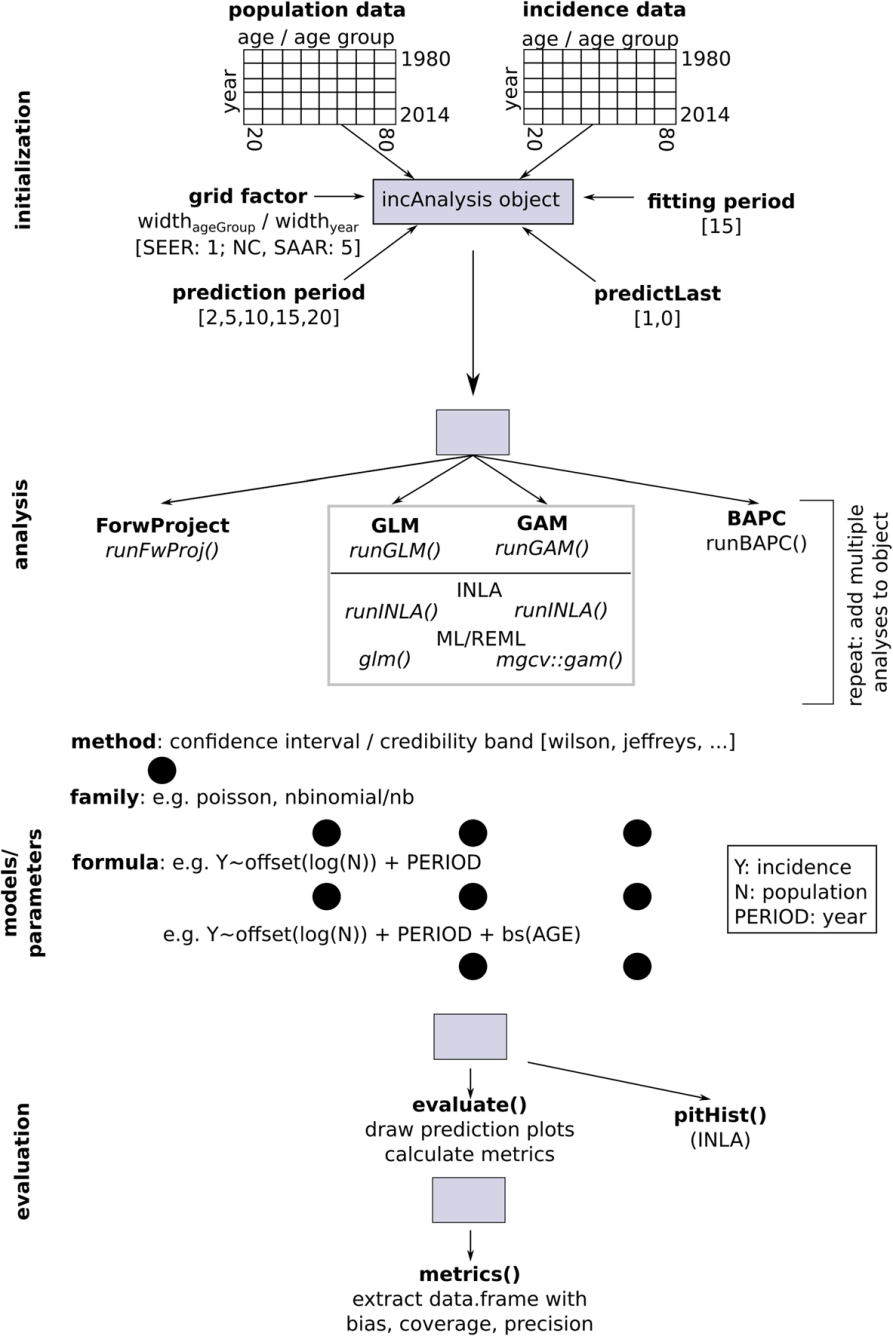
The R package “incAnalysis”

The incAnalysis R package (see details below in section 3.2) was used to evaluate a number of increasingly complex models (GLMs, GAMs, BAPC) using the INLA framework. To describe the evaluated models, we introduce the following notation: *Y* denotes observed cancer incidence counts, *N* denotes population size, *AGE* and *PERIOD* are the respective covariates. The notation also corresponds to variable names used in the R package. Age or age-group, respectively, are indexed by *i*. Selected projections are shown in Suppl.-Figs. 1 and 2.

### Generalized linear models (GLMs)

GLMs are formulated using three components: (1) a probability distribution from the exponential family, (2) a linear predictor *η* = *Xβ* and (3) a link function *g* with *E*(*y*) = *μ* = *g*^−1^(*η*). In all, except BAPC models, negative-binomially distributed counts of tumor cases were assumed.

The most simplistically structured GLM includes only an intercept, *η* = *β*_0_. In R, this intercept-only model was formulated as Y ~ offset (log(N)) (equivalent to: Y ~ 1 + offset (log(N)).

Next, a GLM with age and period as covariates together with their interaction term was assessed: *η* = *β*_0_ + *β*_1_*age* + *β*_2_*period* + *β*_3_*age* : *period*, corresponding to the R formula Y ~ offset (log(N)) + AGE*PERIOD.

### Generalized additive models (GAMs)

GAMs have a structure similar to GLMs, with the difference that smooth functions *f* s of covariates can be included in the linear predictor (*A*: model matrix, *θ*: parameter vector): *g*(*μ*) = *A θ* + *f*_1_(*x*_1_) + *f*_2_(*x*_2_) + ….

Splines might be used as smooth functions, or in the case of INLA, specific Gaussian Markov Random Fields. In the present analysis, B-splines were used as univariate smoother for the age covariate and bs() from the splines package can directly be included in the model formula: Y ~ offset (log(N)) + PERIOD+bs (AGE). Alternatively, an random walk order 2 (rw2) model might be specified as Y ~ offset (log(N)) + PERIOD+f (AGE, model = ‘rw2’).

To allow evaluation of models with multivariate tensor product smoothers for age and period with INLA, we used an ad-hoc solution applying a z-model (we acknowledge that this is a non-standard appraoch and a more detailed outline than in the scope of this article would be useful before more widespread application). Tensor spline interactions can be specified, e.g. by using the function mgcv::te() for the classical model fitting approach (Y ~ offset (log(N)) + te (AGE,PERIOD)). In R-INLA, te() is not directly usable in model formulas. The *z*-model we used instead is an implementation of classical random effects part of a mixed model (*η* = … + *Z z*). The random effects design matrix is $$ \boldsymbol{Z}=\left(\begin{array}{ccc}{\boldsymbol{Z}}_{\mathbf{1}}& \cdots & \mathbf{0}\\ {}\vdots & \ddots & \vdots \\ {}\mathbf{0}& \cdots & {\boldsymbol{Z}}_{\boldsymbol{i}}\end{array}\right) $$ for each cluster *i* which has *q* ∈ *ℕ*^+^ random effects. *Z* was calculated as the tensor product smooth model matrix for marginal bases for age and period model matrices ***A*** and ***P*** using mgcv::tensor.prod.model.matrix() [31]. The *i*th row of the resulting tensor product model matrix is calculated as the Kronecker product of the *i*th rows of ***A*** and ***P***. Marginal bases were calculated as M-splines, using splines2::mSpline() [32]. M-splines are non-negative splines, which can be considered as a normalized version of B-splines. A loggamma prior was specified for this model, with parameter values (a = 1, b = 0.005), the same values used as in [33]. The corresponding R code is shown in the package vignette vignette(‘incidence’).

### Bayesian age-period-cohort models (BAPC)

APC models estimate the individuals’ age, birth cohort and the period in which the event occurred [19]: *η*_*ij*_ = log(*λ*_*ij*_) = *μ* + *α*_*i*_ + *β*_*j*_ + *γ*_*k*_ with intercept *μ*, and age, period and cohort effects *α*_*i*_, *β*_*j*_ and *γ*_*k*_. *i* (1 ≤ *i* ≤ *I*) denotes the age group at time point *j* (1 ≤  *j*  ≤  *j*), the cohort index *k* depends on the age and period index as well as on the age group and period interval width: *k* = *j* + *M* (*I* − *i* ). *M* encodes the width of age groups as compared to period intervals, e.g. for 5 yr age groups and yearly data, *M* is 5. The model implemented in the BAPC package assumes Poisson distributed data, includes the three random effects age, period, cohort (second-order random walk, rw2) and an additional random effect (independent and identically distributed, iid) to adjust for overdispersion. Separate age, period and cohort effects are not identifiable due to the exact linear dependence of effects [19].

### Performance metrics

Model performance was evaluated using three metrics: coverage, bias and precision. Metrics were calculated per age/age-group, sex and entity, and averaged (arithmetic mean), yielding one aggregated value per entity, gender, projection interval and projection models as a summary statistic.

Coverage was calculated as the fraction of projections lying within the 95% (equal tailed) credibility band. Bias was set to 0 if the observed incidence count was equal to the predicted, otherwise the ratio (observed-predicted)/observed was computed. Posterior standard deviations were used as a measure of precision.

### Model performance

Evaluation of the predictive performance of models with increasing complexity was performed as follows (see also Fig. 1): the most current observed incidence data was predicted, with the projection period starting *n* years prior to this timepoint (*n* ∈ {2, 5, 10,15,20}). The observation period for model training preceded this timepoint. In the presented analysis, 15 yrs. were chosen as observation period. For the evaluation of a 2 yr projection, e.g. in the SEER-9 dataset, data of the year 2014 would be predicted, using data from the 15 yrs. prior to 2012 for model fitting.

Das was available in different aggregation types - as age-groups for NORDCAN and Saarland data and for each age for the SEER-9 data. In the latter case, individual age-years were used, i.e. no further aggregation was applied.

### R package incAnalysis

To facilitate further application and reproducibility, the R package ‘incAnalysis’ was developed. It is publicly available on http://github.com/mknoll/incAnalysis. The package mainly builds on methods in the R packages BAPC [19], mgcv [22] and R-INLA [ref: http://www.r-inla.org/]. Representative analyses with stepwise explanations on how to use the package are outlined in the accompanying vignette in more detail: vignette(‘incidence’)in R. An overview of the functionality and structure of the package is given in Fig. 2.

A wide variety of approaches to project future cancer incidence can be comparatively assessed using this package. Constant rates or counts simply projected into the future, as well as GLMs and GAMs (both in the INLA and ML/REML framework, selected via the method parameter) and BAPC models might be specified.

The package provides a class called incClass which is instantiated with population and incidence data (data.frame with years in rows, the earliest available year in the first row and age/age-group as columns with increasing values from left to right) as well as the period used for model training and the fitting period of interest (and additional parameters). Different models are then added to the newly created object with the following functions which usually expect additional parameters, e.g. model formulas and the respective class object: runFwProj() for forward projection of constant rates or constant counts, runGLM() for generalized linear models (using INLA or an ML approach, specified by the method parameter), runGAM() for GAMs, runInla() for any INLA model and runBAPC() to run the BAPC model [19]. evaluate() calculates the performance metrics, which can be extracted as data.frame via metrics(); additionally, projections are plotted. pitHist() plots Probability Integral Transform (PIT) histograms for all INLA fitted models.

## Results

### Coverage

**Fig. 3:**
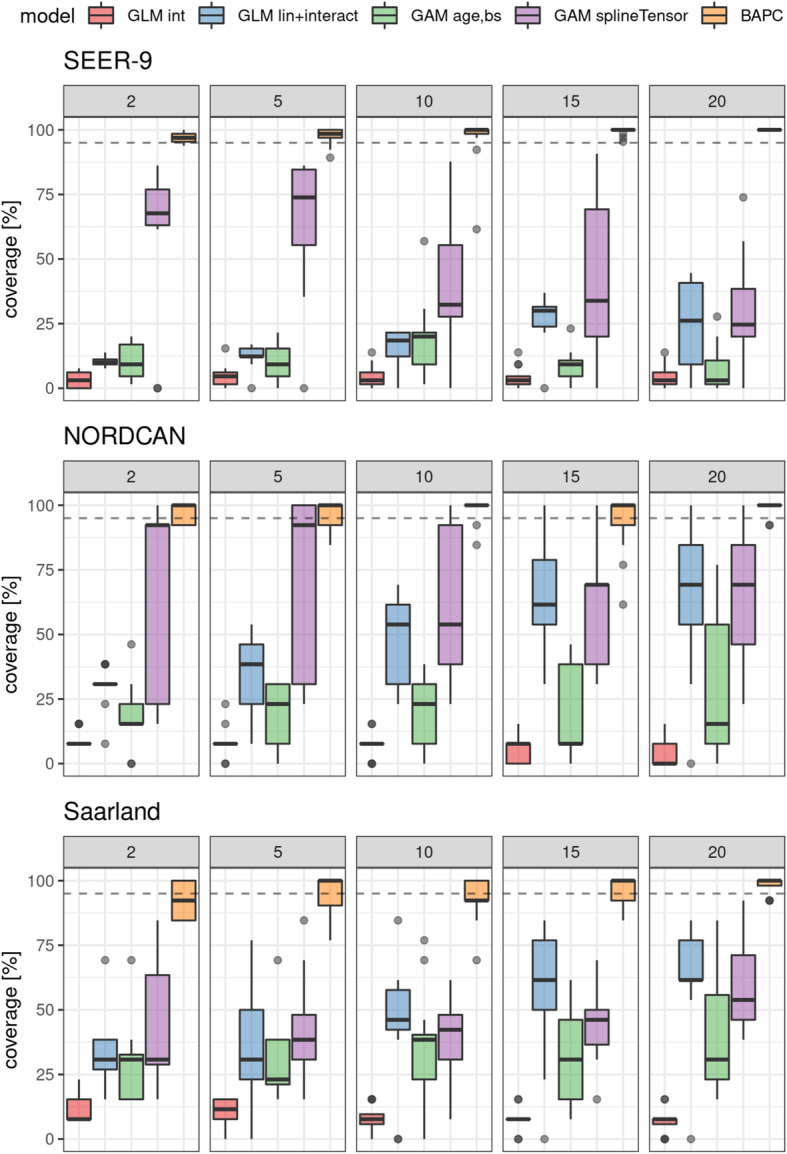
Coverages of future projections after 2, 5, 10, 15 and 20 yrs. based on models with a 15 yr observation period. Dashed line: 95% coverage. Int: intercept only model, lin + interact: linear age, period and interaction effects, age,bs: univariate smoother (B-spline) for age, splineTensor: tensor product smoother (age, period), M-spline basis. GLMs, GAMs: neg-binomial distribution

Coverages for the evaluated models are shown in Fig. 3 for an observation period of 15 yrs. and projection periods of 2, 5, 10, 15 and 20 yrs.

Importantly, most models yielded coverages below 95%, with smallest (< 25%) coverages for intercept only models and highest coverages (> 75%) for BAPC models, irrespective of the projection period. Variability of coverages of BAPC projections is smaller in the SEER-9 dataset as compared to NORDCAN and Saarland data.

Coverage increased for AP models with linear age, period and interaction effect for longer projection intervals in all datasets. Models incorporating a univariate smoother for age showed no clear median increase in coverages for longer periods, variability, however, increased.

Multivariate smoother models showed a decrease of median coverages for longer projection intervals in the SEER-9 data, in increase in the Saarland data and high variability with no clear trend in the NORDCAN data.

### Bias

**Fig. 4:**
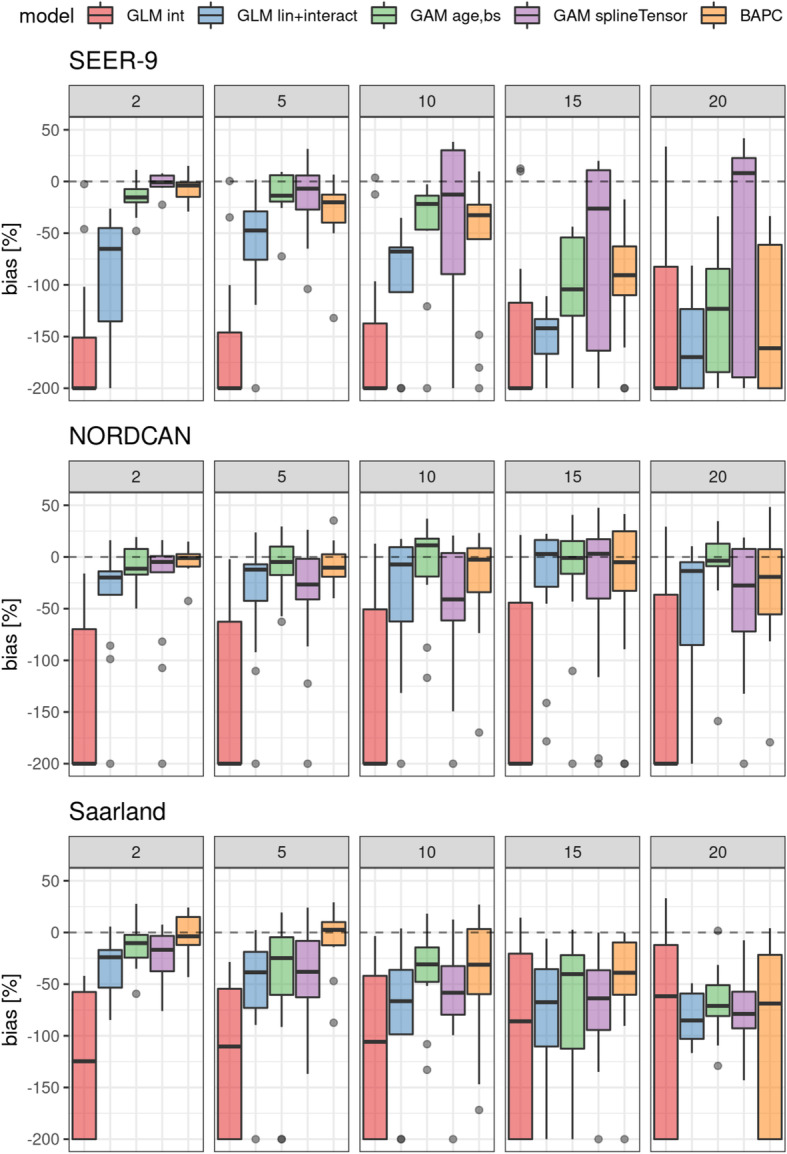
Bias of future projections after 2, 5, 10, 15 and 20 yrs. based on models with a 15 yr observation period. Negative values indicate overestimation of cancer incidence. Bias values smaller than − 200 were set to − 200. Dashed line: no bias (0%). int: intercept only model, lin + interact: linear age, period and interaction effects, age,bs: univariate smoother (B-spline) for age, splineTensor}: tensor product smoother (age, period), M-spline basis. GLMs, GAMs: neg-binomial distribution

Results of bias analyses are shown in Fig. 4. Negative values correspond to higher predicted than observed incidence counts (overestimation). For visualization purposes, values <− 200 were set to − 200.

Several models show negative values. Absolute bias increases with longer projection intervals for most models in the SEER-9 and Saarland datasets. Intercept-only models show mostly absolute median bias values below − 100, except for 15 and 20 yr projections in the Saarland data. Univariate smoother models show in most cases lower absolute bias as GLMs with linear age, period and interaction effects. Median absolute bias is smallest for the multivariate smoother models in SEER-9 data for longer projection intervals. Differences in median absolute bias between all except intercept-only models are highest in the SEER-9 dataset.

### Precision

**Fig. 5:**
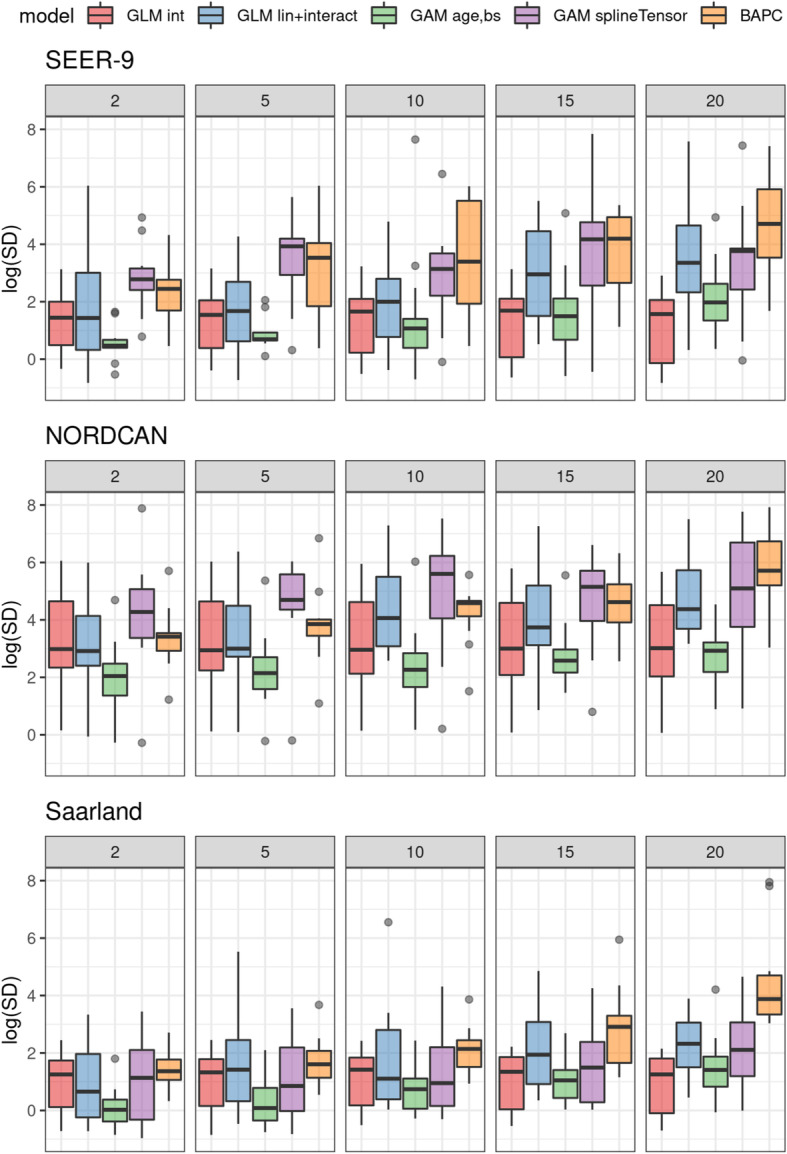
Precision of future projections after 2, 5, 10, 15 and 20 yrs. based on models with a 15 yr observation period. Transformed averaged posterior standard deviations are shown. Int: intercept only model, lin + interac: linear age, period and interaction effects, age,bs: univariate smoother (B-spline) for age, splineTensor: tensor product smoother (age, period), M-spline basis. GLMs, GAMs: neg-binomial distribution

Precision is depicted in Fig. 5; median model values range mostly between 0.5 and 5 for the SEER-9 data, 2 and 6 for the NORDCAN data and 0 and 4 in the Saarland dataset. Longer projection intervals yield lower precision for all but the intercept only model. Univariate smoother models show higher precision as compared to most additionally evaluated models. Variability in precision increases for longer projection intervals for the BAPC models, and for the SEER-9 data, for univariate smoother GAMs. For the other models, no clear trend can be observed.

## Discussion

Population-based cancer registry data are routinely used to monitor cancer incidence at the population-level, to evaluate screening and prevention programs, and to identify areas where intensified medical research is needed [4]. However, no consensus appears to exist on which models to use for projections based on short-term observational rate data in cancer epidemiology. Systematic empirical evaluations of potentially applicable approaches using existing cancer registry data for benchmarking appear sensible to obtain a better understanding of their operating characteristics and to ultimately make informed methodological recommendations. To facilitate this idea, we introduced an R package (incAnalysis) for an integrated evaluation of the adequacy of different statistical approaches in this context. We note that the package could in principle also be used for projections of other types of rates than incidence rates. In an extensive and systematic evaluation we demonstrated its use. While the presented results may already be informative for methodological guidance, we believe that further detailed and targeted applications would be helpful for the derivation of methodological guidance by expert panels. Consensus on desirable (or acceptable) operating characteristics would be sensible prerequisite for the appraisal of individual statistical modeling options.

In the reported empirical analysis only age(−groups) between 20 and 84 were analyzed, as childhood tumors constitute a biologically distinct group, are in general rare and require reliable projections of birth rates. This might impair the ability of models to obtain reliable projections; nevertheless it has been reported [34] that this approach might decrease accuracy. Cancers in the age group > = 85 were excluded to assure comparability between cancer registries (fixed age-group width required for BAPC [gridFactor]).

Model performance was assessed by evaluating coverage, bias and precision of projections. Alternative metrics for model evaluation described are e.g. the Continuous Ranked Probability Score (CPRS) as used e.g. in [19] or the evaluation of PIT histograms. The latter can be easily obtained from INLA fitted objects, and further metrics as the CPRS can be easily calculated using the data provided by the incAnalysis package.

As the least complex model, intercept only models were evaluated. As expected, only small coverages (< 25%) could be expected as cancer occurrence is usually highly dependent on age. An intercept only model does not take the age into account (change in the distribution of age over calendar time), and thus, these models can hardly be recommended for cancer incidence projection, especially over a longer period.

GLMs with linear age, period and their interaction effect were evaluated as next, more complex model types. Performance, however, was generally poor. To achieve a potentially even better fit, a model with a univariate smoother for age was analyzed, as the latter is a biologically highly relevant covariate for cancer incidence. B-splines, created with splines::bs() were incorporated into the model formula. An alternative would be the specification of a Gaussian Markov Random Field structure for smoothing, e.g. a second order random walk.

Next, multivariate smoothers (tensor product smoothers) for age and period were included into the model, using a *z*-model in INLA. For classical ML/REML models, such effects can easily be included in the models by using the mgcv::te() function. The latter cannot be directly fit with INLA::inla(). Even though the mgcv::ginla() function was made available recently (which allows to obtain posterior distributions of effects directly from GAMs fitted with mgcv), the INLA package is not directly utilized by mgcv, and thus projections are not as straight-forward as with the *z*-model. Coverage is higher as compared to univariate smoother models, but is less stable for long term projections as compared to BAPC models.

Finally, the BAPC model was evaluated and turned out to be among the best performing for all evaluated parameter combinations. The additional two effects (cohort and overdispersion adjustment effect) seem to be especially important for short-term projections, as differences to most other models except multivariate smoother models decrease for longer intervals.

## Conclusions

The incAnalysis R package allows a straightforward comparison of key operating characteristics of statistical approaches to cancer incidence projection. Our empirical analyses of a selection of potentially applicable approaches suggest that (i) projections of rate data using short term data yields robust high coverage at the cost of low precision for BAPC, (ii) age-period GLMs with interaction term mostly yield better results for longer projection intervals (> 10 yrs), (iii) GAMs using tensor product smooth models (age, period) constitute a reasonable alternative to classical GLMs, and (iv) intercept-only models may at best be useful only for short-term projections (< 5 yrs). Further detailed and targeted investigations into model performance seem advisable to make recommendations about appropriate statistical projection methods in a given setting.

## Supplementary information

**Additional file 1.**

**Additional file 2.**

**Additional file 3.**

## Data Availability

The datasets generated and/or analysed during the current study are included in the incAnalysis github package, https://github.com/mknoll/incAnalysis.

## Abbreviations

APC: Age-Period-Cohort
BAPC: Bayesian APC models
CPRS: Continuous Ranked Probability Score
GAM: Generalized Additive Model
GLM: Generalized Linear Model
INLA: Integrated Nested Laplace Approximations
MCMC: Markov-Chain Monte Carlo
ML: Maximum Likelihood
PIT: Probability Integral Transform
REML: Restricted Maximum Likelihood
