# Supplementary material for: An R package for an integrated evaluation of statistical approaches to cancer incidence projection
Source: BMC Med Res Methodol. 2020 Oct 15;20:257. doi: 10.1186/s12874-020-01133-5 (PMC7559591; doi:10.1186/s12874-020-01133-5)
Supplement: Supplementary file 1 — Additional file 1. [file 12874_2020_1133_MOESM1_ESM.docx]

**Supplementary Table 1:** Structure of incidence and population data. Brain incidence data (males) from the NORDCAN cancer registry. Age-group in columns, years in rows.

| **incidence** | | | | | | | | | | | | | |
| --- | --- | --- | --- | --- | --- | --- | --- | --- | --- | --- | --- | --- | --- |
|  | **20** | **25** | **30** | **35** | **40** | **45** | **50** | **55** | **60** | **65** | **70** | **75** | **80** |
| **2011** | 40 | 67 | 90 | 117 | 144 | 186 | 234 | 242 | 308 | 340 | 267 | 200 | 137 |
| **2012** | 58 | 69 | 98 | 126 | 142 | 193 | 224 | 254 | 294 | 354 | 281 | 207 | 135 |
| **2013** | 53 | 71 | 74 | 106 | 135 | 170 | 212 | 267 | 238 | 396 | 260 | 217 | 142 |
| **2014** | 76 | 76 | 95 | 125 | 125 | 168 | 213 | 253 | 299 | 367 | 288 | 220 | 136 |
| **2015** | 49 | 63 | 83 | 102 | 121 | 171 | 199 | 208 | 306 | 326 | 309 | 209 | 133 |
| **population** | | | | | | | | | | | | | |
|  | **20** | **25** | **30** | **35** | **40** | **45** | **50** | **55** | **60** | **65** | **70** | **75** | **80** |
| **2011** | 847786 | 811124 | 818311 | 869784 | 907205 | 923488 | 843973 | 817177 | 832029 | 709493 | 489595 | 354109 | 253143 |
| **2012** | 871118 | 824001 | 823642 | 861435 | 895604 | 940211 | 846714 | 818951 | 809814 | 747214 | 504623 | 356150 | 250076 |
| **2013** | 888281 | 840394 | 832062 | 856515 | 887625 | 946472 | 857464 | 819322 | 797309 | 777677 | 527760 | 638781 | 251811 |
| **2014** | 896594 | 864154 | 841734 | 854332 | 88649 | 940717 | 875771 | 821564 | 787670 | 792512 | 558810 | 384445 | 253975 |
| **2015** | 902694 | 896041 | 853876 | 851729 | 891603 | 925494 | 899530 | 826467 | 783202 | 790762 | 602674 | 400912 | 258915 |
