# Supplementary figures and images for: An R package for an integrated evaluation of statistical approaches to cancer incidence projection
Source: BMC Med Res Methodol. 2020 Oct 15;20:257. doi: 10.1186/s12874-020-01133-5 (PMC7559591; doi:10.1186/s12874-020-01133-5)

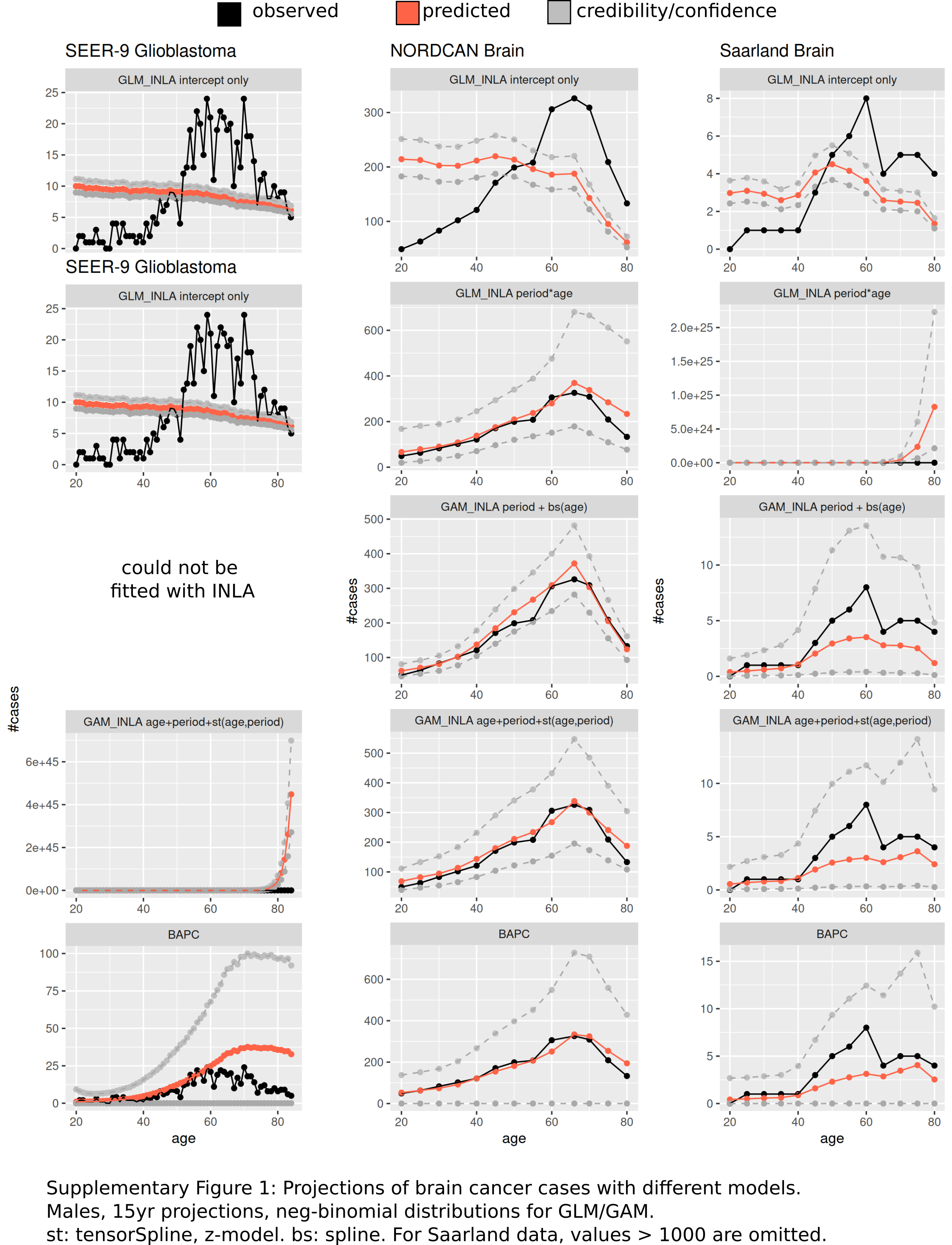

Supplement: Supplementary file 2 — Additional file 2. [file 12874_2020_1133_MOESM2_ESM.png]

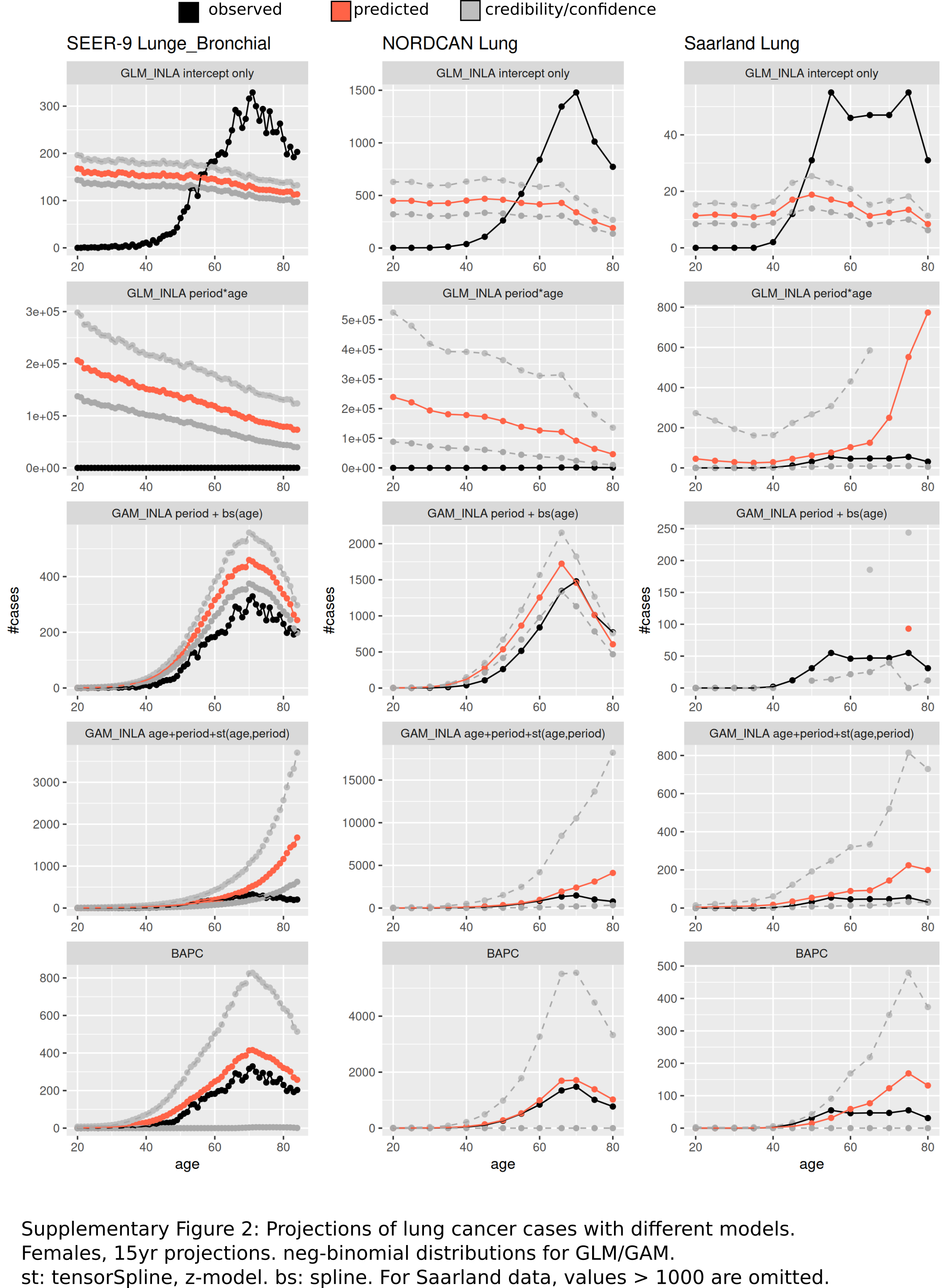

Supplement: Supplementary file 3 — Additional file 3. [file 12874_2020_1133_MOESM3_ESM.png]
